# Using Stereochemistry to Control Mechanical Properties in Thiol–Yne Click‐Hydrogels

**DOI:** 10.1002/ange.202107161

**Published:** 2021-10-28

**Authors:** Laura J. Macdougall, Maria M. Pérez‐Madrigal, Joshua E. Shaw, Joshua C. Worch, Christopher Sammon, Stephen M. Richardson, Andrew P. Dove

**Affiliations:** ^1^ Department of Chemistry University of Warwick Coventry CV4 7AL UK; ^2^ School of Chemistry University of Birmingham Birmingham B15 2TT UK; ^3^ Division of Cell Matrix Biology and Regenerative Medicine School of Biological Sciences Faculty of Biology, Medicine and Health Manchester Academic Health Science Centre University of Manchester Manchester M13 9PL UK; ^4^ Materials and Engineering Research Institute Sheffield Hallam University Sheffield S1 1WB UK

**Keywords:** cellular mechanoresponsive behaviour, click-chemistry, hydrogels, stereochemistry, thiol–yne nucleophilic addition

## Abstract

The stereochemistry of polymers has a profound impact on their mechanical properties. While this has been observed in thermoplastics, studies on how stereochemistry affects the bulk properties of swollen networks, such as hydrogels, are limited. Typically, changing the stiffness of a hydrogel is achieved at the cost of changing another parameter, that in turn affects the physical properties of the material and ultimately influences the cellular response. Herein, we report that by manipulating the stereochemistry of a double bond, formed in situ during gelation, materials with diverse mechanical properties but comparable physical properties can be obtained. Click‐hydrogels that possess a high % trans content are stiffer than their high % cis analogues by almost a factor of 3. Human mesenchymal stem cells acted as a substrate stiffness cell reporter demonstrating the potential of these platforms to study mechanotransduction without the influence of other external factors.

## Graphical abstract

Controlling the stereochemistry of double bonds that are formed in situ using the nucleophilic thiol–yne addition enables the synthesis of robust hydrogels with mechanical properties that relate to the double bond stereochemistry. This approach allows the synthesis of hydrogels in which the other chemical and hence physical parameters remain the same providing a platform from which to isolate mechanical properties to direct cell differentiation.

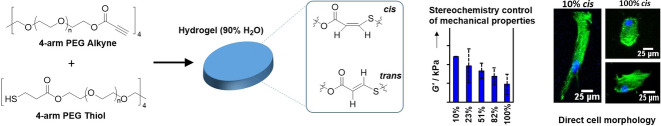

## Introduction

The 3‐dimensional arrangement of bonds, or stereochemistry, dictates the function and behaviour of molecules spanning from biological systems to drugs to synthetic polymers. Stereochemistry is critical in the production of the basic building blocks of life. The chirality of deoxyribose dictates the backbone structure of DNA, enabling the formation of a double helix structure which is critical for biological processes in living organisms. The power of stereochemistry is also apparent in small molecule chemistry in which it can be critical in the biological behaviour of the resulting compound. Differences in bond orientation also significantly affect the bulk material properties of polymers,^[1]^ where the relative stereochemistry of pendant groups (tacticity) in synthetic polymers is most commonly used to control their properties.^[2]^ In another example, naturally occurring geometric isomers of polyisoprene lead to significant differences in materials' properties depending on the *cis*–*trans* configuration. The *cis* isomer of high molecular weight polyisoprene is the base component of elastic natural rubber, but the *trans* isomer is a more crystalline and much harder material, known as Gutta Percha.^[3]^

While it is well known that control over stereochemistry in polymers results in notable changes in thermal and/or mechanical properties,^[1]^ the use of such a concept to significantly influence a highly swollen network has been comparatively less studied. A few reports describe the *cis*:*trans* isomerism of the photo‐switchable azobenzene^[4]^ moiety to change mechanical properties of hydrogels,^[5]^ although the observable differences in properties are modest and transient as a consequence of reversion to the thermodynamically favoured *trans* isomer. Hence, the ability to finely tune hydrogel mechanical properties remains an important objective in biomaterial engineering because 1) hydrogels already provide a controlled platform to understand how cells interact with their surroundings[^5c^, ^6^] and 2) it is well‐established that cells sense and integrate mechanical cues from the native extracellular matrix (ECM), which ultimately directs gene expression and cell‐fate decisions.^[7]^

Several hydrogel‐based approaches have been explored to mimic the ECM and thus better understand biological processes, including aging, injury, and disease progression.^[8]^ Inspired by the native dynamism of the cell mechanical microenvironment,^[9]^ early work in this area investigated the effect of hydrogel degradation on cell behaviour;^[10]^ however, this process is inherently challenging to control because of issues related to swelling and cytocompatibility concerns.^[11]^ More recently, a range of different hydrogel materials have been examined to further understand mechanobiology, matrix deposition, and disease progression by creating systems with tuneable viscoelastic properties.^[12]^ Photochemical pathways have also been explored as a means to stiffen and/or degrade hydrogels.[^12d^, ^13^] Finally, several dynamic systems featuring stiffness reversibility in response to external stimuli (e.g. pH,^[14]^ temperature,^[15]^ host–guest interactions,^[16]^ or salt concentration^[17]^) have similarly been reported. However, hydrogel stiffness, or elasticity, is typically modulated by altering the fundamental material formulation. This includes changes to the molecular weight of the polymer precursors, the overall polymer content in the hydrogel, or the degree of crosslinking. However, variations in formulation often manifest in changes in the physical nature of the network,^[18]^ such as pore size/distribution, swelling behaviour or degradation profile. Since these parameters are inherently linked to the chemistry of the network as it forms, decoupling their effect from each other is challenging.^[19]^

**Scheme 1:**
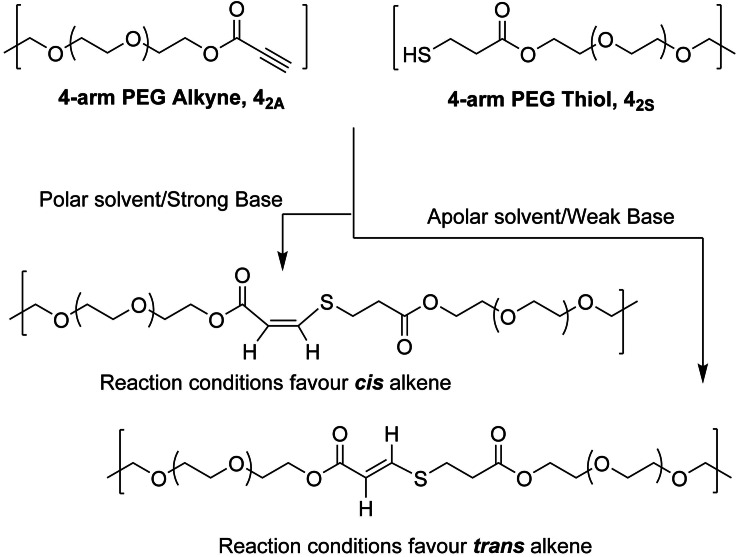
The synthesis of thiol–yne click‐hydrogels with controllable alkene stereochemistry by adjusting reaction parameters. Click‐hydrogel architecture used in the study: alkyne‐ and thiol‐functionalised PEG precursors, **4_2A_** and **4_2S_** in which the main number relates to the number of arms on the PEG precursor, 4‐arm, while the subscript denotes the molecular weight of the polymer and functional group (2 kg mol^−1^, alkyne (A) or thiol (S)).

The nucleophilic thiol–yne addition reaction yields unsaturated polymers where the stereochemistry of the alkene product can be readily controlled through solvent polarity and base strength.^[20]^ Briefly, when using apolar solvents and/or a weak base the *trans* isomer is favoured, while the *cis* isomer is favoured in polar and/or strong base reaction conditions (Scheme 1). Previous work in our group has utilised this reaction for the synthesis of thermoplastic materials with tuneable mechanical properties defined by the stereochemistry of the alkene moiety.^[21]^ Furthermore, we have synthesised nucleophilic thiol–yne addition click‐hydrogels as robust ECM mimics which can encapsulate cells and modulate stiffness and swelling.^[22]^

Herein, we show that the effect of stereochemistry within the swollen click‐hydrogel networks (ca. 90 % water) influences the bulk mechanical properties of the resulting materials. Specifically, we demonstrate how controlling stereochemistry enables discrete changes in mechanical strength and substrate stiffness, without changing other physical properties that influence cell response (i.e. crosslinking density, pore size or toxicity). Hence, our click‐hydrogels can contribute to understanding how cells are influenced by stiffness alone, and, how cells sense different polymer stereochemistry.

## Results and Discussion

To exploit the benefits of using the nucleophilic thiol–yne addition reaction to create stererochemically defined robust click‐hydrogels, we chose the PEG‐based click‐hydrogel **4_2A_4_2S_** (in which the main number relates to the number of arms on the alkyne (A) or thiol (S) precursor, while the subscript denotes the molecular weight of the polymer and functional group; Scheme 1, Figure S1) as our model since it had exhibited the highest compressive strength (in the order of 0.1–0.4 MPa) over time with little swelling.^[22b]^ Indeed, because of the hydrophobic nature of the crosslinked network, no bulk degradation occurred for 15 days after immersing the click‐hydrogels in an aqueous environment under physiological conditions. Alkyne‐ and thiol‐functionalised PEG precursors, **4_2A_** and **4_2S_** (4‐arm, 2 kg mol^−1^), respectively, were synthesised as reported previously by a highly efficient Fischer esterification.^[22b]^ Optically transparent **4_2A_4_2S_** gels were prepared with a solids content of 10 wt % by mixing solutions containing a 1:1 molar ratio of alkyne to thiol polymer precursors at ambient temperature using different solvent mixtures to tune the final stereochemistry of the crosslinked network. Based on previous work, different solvents with various polarities were used to control the stereochemistry.^[20b]^ Specifically, two from three solvents (i.e. CHCl_3_ (non‐polar), acetone (moderately polar), and H_2_O (polar)) were mixed in different ratios (Table 1), while triethylamine (NEt_3_) acted as the catalyst for the thiol–yne click chemistry reaction. The amount of triethylamine added to each gel‐precursor solution, which was adjusted based on the solvent polarity, so that the gelation time for all the gels was ca. 1 minute regardless of the final *cis*:*trans* ratio in order to retain comparable network parameters. After mixing the precursors, the organogels were cured at ambient temperature for 1 h to ensure that the reaction had proceeded to completion. Neither the polarity of the solvent nor the amount of base used affected the gel fraction (GF) values, which were higher than 92 % for all the systems. This evidences the high efficiency of the crosslinking reaction and comparability of the fundamental gel structure across all formulations.

**Table 1:** Gelation conditions (solvent and amount of catalyst) used to prepare gels with varying *cis:trans* ratio.^[a]^

Solvent (v/v)	[NEt_3_] [μL mL^−1^]	% *cis* content^[b]^	Gel fraction [%]	EWC^[c]^ [%]	Mesh size [nm]	*T*_g_^[d]^ [°C]	*T*_cc_^[d]^ [°C]	*T*_m_^[d]^ [°C]	Δ*H_m_* ^[d]^ [J g^−1^]
H_2_O	0.7	100	97±1.0	92.1±0.6	6.7±0.2	−42.7	−13.5	19.0	15.8
H_2_O:Acetone 35:65	1.66	82	92±2.0	88.0±0.4	5.4±0.1	−43.8	−10.3	16.8	16.6
H_2_O:Acetone 10:90	10	51	95±1.5	89.3±0.2	5.6±0.2	−44.5	−10.9	19.0	13.3
Acetone:CHCl_3_ 60:40	20	23	94±1.0	87.4±0.1	5.2±0.1	−45.5	−11.6	15.5	12.2
CHCl_3_	34	10	96±1.0	84.5±0.3	4.5±0.1	−48.8	−23.4	19.7	18.0

[a] All PEG‐based click‐hydrogels were synthesised using **4_2A_** (4‐arm alkyne, 2 kg mol^−1^) and **4_2S_** (4‐arm thiol, 2 kg mol^−1^) at 10 wt % solids content. [b] Determined by FT‐IR spectroscopy *n*=3 (12 runs/gel). [c] Equilibrium water content. [d] Thermal data obtained from DSC thermograms. *T*_g_=glass transition temperature, *T_cc_* =cold‐crystallisation temperature, *T_m_* =melting temperature, Δ*H_m_* =melting enthalpy.

**Figure 1:**
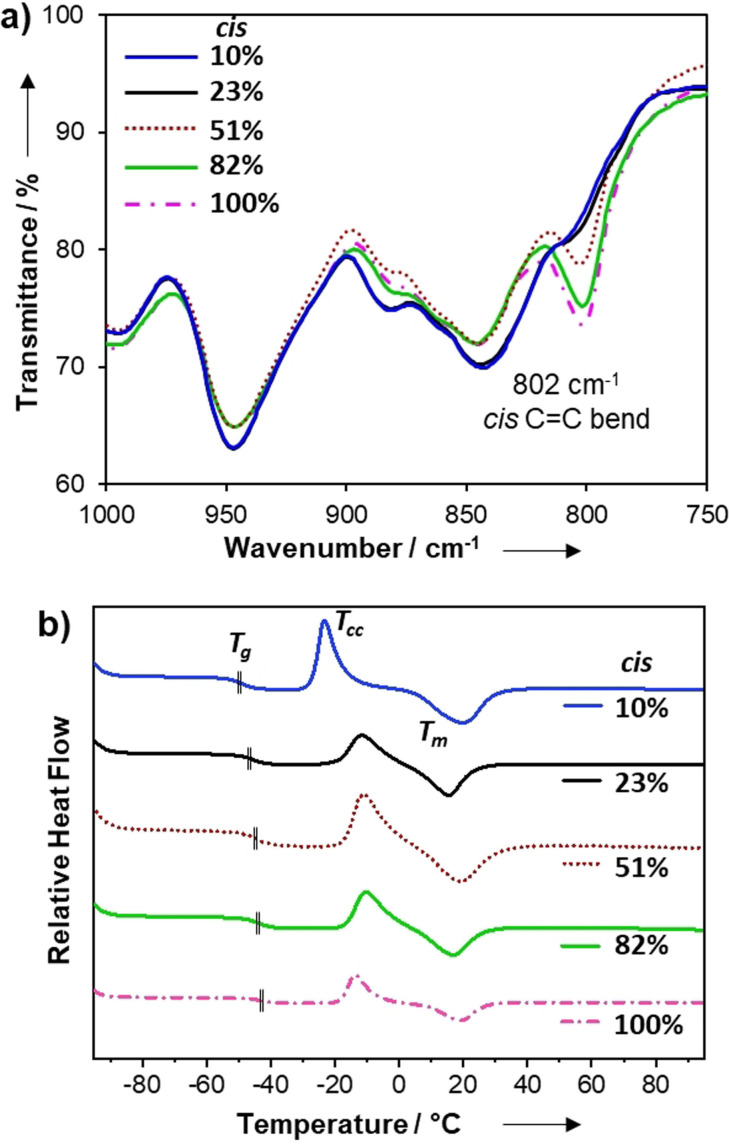
a) FT‐IR spectra obtained for stereochemically controlled PEG‐based click‐hydrogels *n*=3 (12 runs/gel). b) DSC thermograms (endo up) of 2nd heating cycle for dried gels tested at 10 °C min^−1^. Position of *T*_g_ for each sample is indicated with a vertical hash mark.

The *cis*:*trans* content in the gels was determined by FT‐IR spectroscopy of the dried samples using the diagnostic signal attributed to the *cis* C=C bend at 802 cm^−1^, which was largely absent in the low *cis* materials (Figure 1 a, see full experimental details in the Supporting Information; Figure S2). These studies indicate a large range in stereochemical composition (10, 23, 51 and 82 % *cis* content), which suggests that the polarity of the reaction medium was positively correlated to *cis* content which is in accordance with a previous report.^[20b]^ Small molecule model studies using monofunctional thiols and PEG‐propiolates enabled NMR spectroscopic analysis of the double bond stereochemistry and also indicated that an analogous relationship between reaction polarity and *cis* content existed (Figure S3–S6). However, the stereochemical content of the products appeared sensitive to reaction conditions, such as temperature or concentration, and sometimes varied between repeated trials. Nevertheless, the stereochemistry of the gels appeared less sensitive to the experimental conditions and consistent values from FT‐IR spectra were observed among repeated formulations. Hence, from herein onwards, % *cis* content of the gels is referred to from the values determined by FT‐IR spectroscopy of the isolated gels.

After drying the materials to control for solvent effects, we studied the thermal properties of the stereochemically defined networks by using differential scanning calorimetry (DSC). In addition to a glass transition (*T*_g_), all formulations also displayed a pronounced cold‐crystallisation (*T*_cc_) event followed by a first‐order melt transition (*T*_m_) (Figure 1 b). This crystallisation/melting behaviour was unexpected and suggests that the materials were highly ordered, despite their crosslinked architecture. Although the overall thermal profile of each dried gel is comparable, small differences can be related to stereochemistry. For example, the *T*_g_s ranged from −43 to −49 °C, increasing with *cis* content of the material (Table 1 and Figure 1 b; Supporting Information Figures S7–S12). Intuitively, the materials with mixed stereochemistry (23 % and 51 % *cis* samples) had the lowest crystallinity (i.e. more disorder) as evidenced by their comparatively low enthalpy of melting (Δ*H_m_*), and this increased slightly for the high *cis* gels (82 % and 100 % *cis*, Figure S12). However, the low *cis* gel (10 % *cis*) had a relatively low *T*_cc_ (Δ*T*_cc_≥10 °C) and the highest Δ*H_m_* among the materials, which indicates that it is the most crystalline. These data suggest that bulk ordering of the material is increased by stereochemical purity (high or low *cis* content), although the 10 % *cis* gel affords noticeably higher crystallinity, likely from better chain‐packing due to the favourable conformation of the *trans* alkene.

In order to study the properties of comparable click‐hydrogel materials, organogels were transitioned into water by initial immersion in acetone for 5 d with frequent solvent changes before gradual introduction of water into the network over 2 d. Acetone was chosen since it is miscible with both CHCl_3_ and H_2_O, as well as the triethylamine catalyst and, importantly, minimal hydrolysis was expected during either step. Notably, this procedure was carried out for all gels to ensure comparability of the end product and remove uncertainty that may arise from the process. Measurement of the post‐washed equilibrium swelling content (EWC) revealed that regardless of the % *cis* content, the EWC was higher than 84 % (Table 1). Indeed, similar EWC values (i.e. between 86 % and 95 %) were determined for a series of thiol–yne PEG click‐hydrogels prepared in PBS, and thus with high % *cis* content,^[22b]^ which indicates that the differences regarding solvent choice, and in turn stereochemistry, did not significantly affect the ability of the PEG‐based thiol–yne click‐hydrogels to hold water.

The mesh size of the range of click‐hydrogels was calculated using the Flory–Rehner equation (See Supporting Information). This parameter of the stereochemistry‐controlled gels varied from 4.5 nm (10 % *cis*) to 6.7 nm (100 % *cis*), with the intermediate systems displaying an average mesh size of 5.4 nm (Table 1)—which is a very small difference for click‐hydrogels over such a range of stiffness.[^18c^, ^23^] Further studies based on Fluorescence Recovery after Photobleaching (FRAP) also showed the same trend (Supporting Information; Figure S13 and Table S2). In this case, the mesh size was estimated to be smaller, ca. 2.5 nm, for the CHCl_3_ system (10 % *cis*) than for the H_2_O‐based system (100 % *cis*, ca. 4.0 nm). Hence, we observe some correlation between the mesh size and the *cis*:*trans* ratio for the extreme conditions, which we ascribe to the packing of the polymeric network. Most likely, the slightly smaller mesh size for the lowest *cis* material is a consequence of the increased crystallinity displayed by this sample under DSC analysis. However, the general trend was that high % *trans* configurations of the vinyl thioether double bond favoured a more ordered and dense arrangement of the PEG chains, which then resulted in a smaller mesh size.

**Figure 2:**
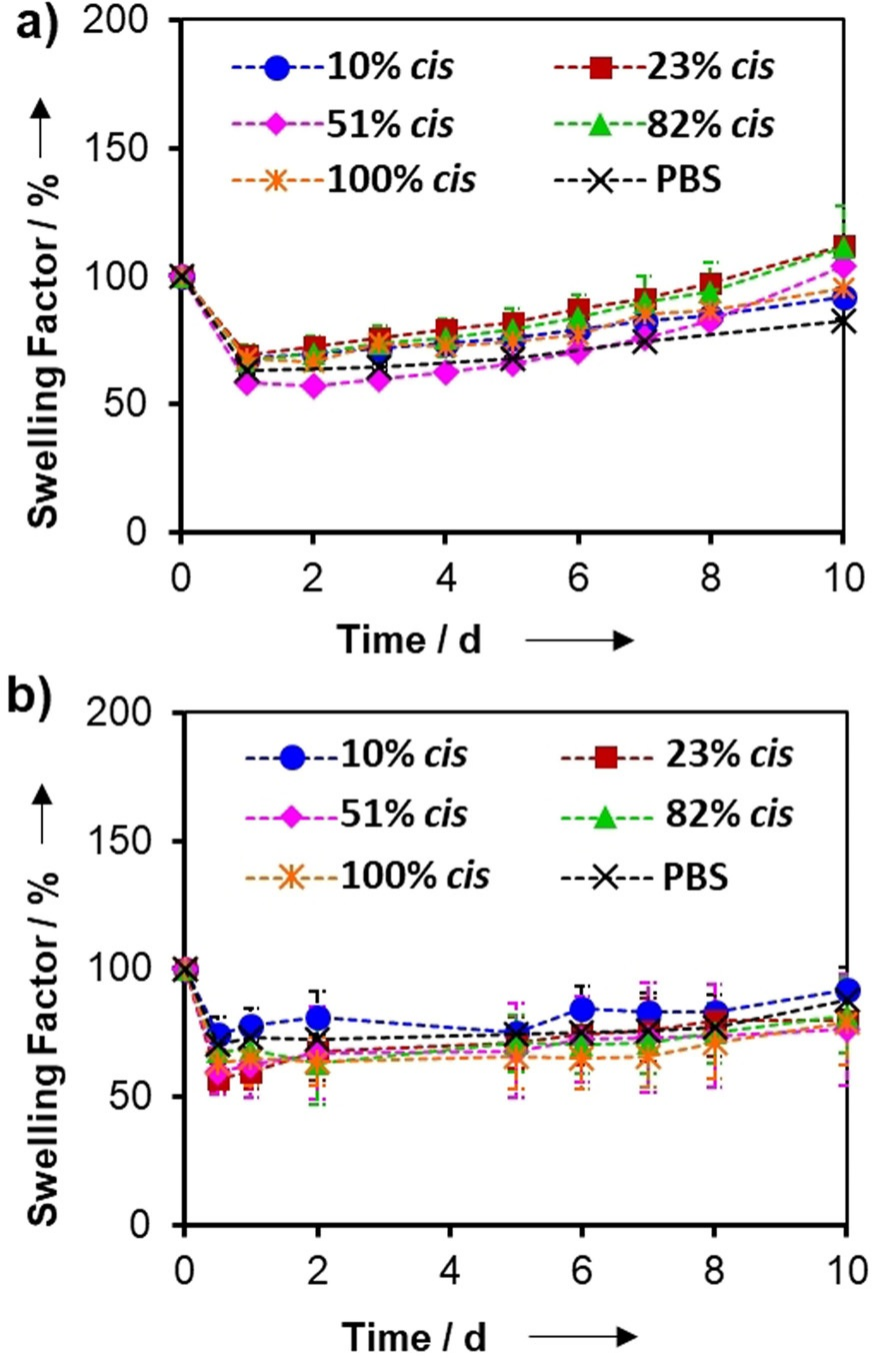
a) Swelling factor (SF) profiles of stereochemically controlled click‐hydrogels immersed in a) PBS and b) cell culture media at 37 °C and mild shaking, *n*=3.

Of major importance to the application of these click‐hydrogels as 2D platforms for cell culture studies is the swelling profile of the click‐hydrogels. As such, this was monitored over time after their immersion in PBS solution at 37 °C (Figure 2 a). The stability of the click‐hydrogels, as well as their mechanical integrity, was retained when immersed in an aqueous environment. Specifically, all click‐hydrogels, irrespective of their stereochemistry, initially shrank down to ca. 60 % of their original mass when placed in PBS, before swelling back slowly to their original weight (Swelling factor (SF) of 100 %) within 10 d, after which the hydrolysis of the ester linkages proceeded. This action decreased the structural integrity of the material, evidenced by the increased SF values and final dissolution of the click‐hydrogels within 30 d. The click‐hydrogels showed a similar response when immersed in cell culture media (Figure 2 b). All click‐hydrogels exhibited a similar degradation profile that was comparable with hydrogels synthesized in PBS, which demonstrates that the thiol–yne click chemistry is highly adaptable, forming virtually the same PEG network regardless of the solvent polarity or catalyst used. Therefore, the control over the stereochemistry within the polymeric network has a minimal effect on the swelling behaviour and stability of these materials, which ensures their potential for biological applications, such as robust platforms for short‐term cell culture studies.

**Figure 3:**
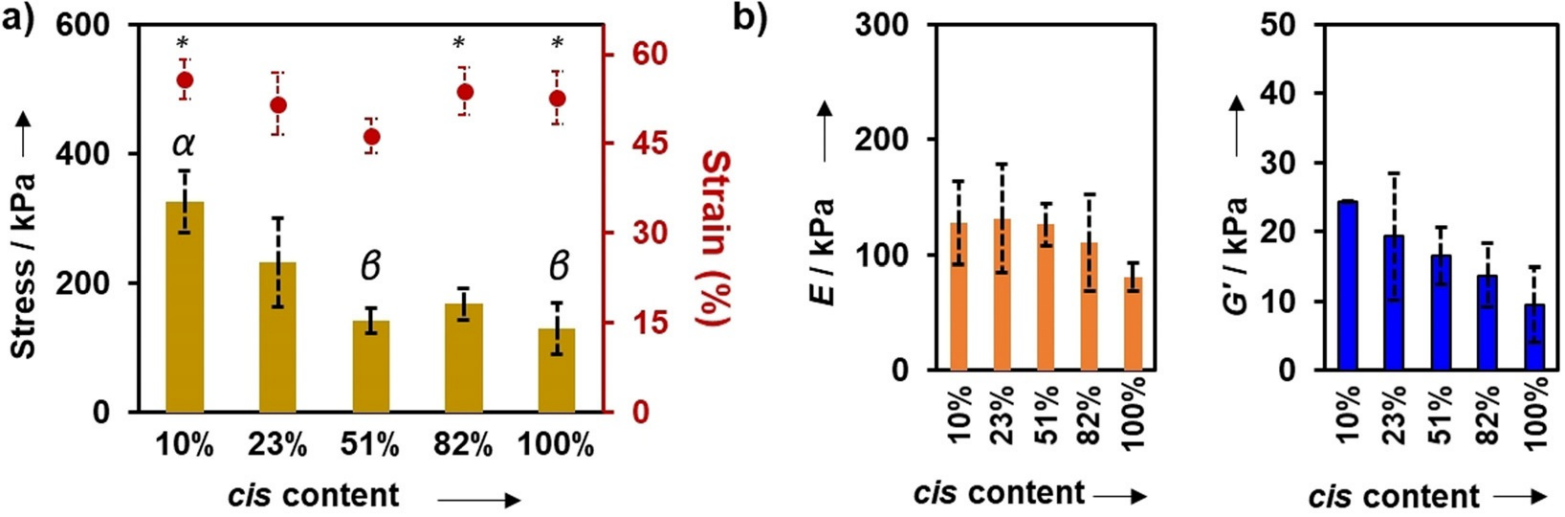
Mechanical characterisation of stereochemically controlled click‐hydrogels [Error bars: SD with *n*=7–9]. a) Compressive strength (bars, left axis) and strain at break (circles, right axis) values; Greek letters on the bars refer to significant differences (*p*‐value<0.05): α vs. all; β vs. 23 %; Symbols on the circles refer to significant differences (*p*‐value<0.05): * vs. 51 %. b) Stiffness defined as compressive Young's modulus (left graph) and storage modulus (*G*′ at 0.1 % strain, refer to Figure S14b).

Given the similarities in physical behaviour that comes only from the stereochemistry being altered between click‐hydrogels, we were eager to understand how the stereochemistry could affect the mechanical properties of the gels. In a previous report describing linear elastomeric polymers, the Young's modulus and ultimate tensile strength were shown to vary by an order of magnitude according to *cis* content,^[21b]^ which presented the potential that these gels may also have a large difference in properties. The mechanical properties of the click‐hydrogels were assessed through uniaxial compression and rheological testing (Figure S14). For compression testing, cylindrical click‐hydrogels (4 mm in height × 9 mm in diameter) were subjected to compressive loading until 98 % strain. Regardless of the % *cis* content, all click‐hydrogels failed within 46–56 % of strain, while the stress at break (i.e. compressive strength) varied according to the % *cis* content (Table 2, Figure 3). Specifically, **4_2A_4_2S_** click‐hydrogels with high % *cis* content exhibited significantly lower compressive strength (130±40 kPa) in comparison to low % *cis* content click‐hydrogels, which could withstand significantly higher amounts of compressive load (326±49 kPa). Similarly, click‐hydrogels with 10 % *cis* content showed a compressive Young's modulus 58 % higher than that displayed by click‐hydrogels with 100 % *cis* content (i.e. 128 kPa and 81 kPa for 10 % and 100 % *cis* content, respectively). Hence, as expected, the stereochemistry of the PEG networks influences the mechanical response of the click‐hydrogels. Indeed, the relative conformation ratio of the *cis* and *trans* vinyl thioether crosslinks correlates with the bulk mechanical properties of the materials, the **4_2A_4_2S_** click‐hydrogels with low % *cis* content being stiffer and more robust.

**Table 2:** Mechanical properties determined for the stereocontrolled thiol–yne PEG click‐hydrogels by uniaxial compression testing and rheological characterisation.

% *cis*	Strength at break [kPa]^[a]^	Strain at break [%]^[a]^	*E* [kPa]^[a,b]^	*G*′ [kPa]^[c]^
10	326±49	56±3	128±36	24.3±0.1
23	232±68	52±5	132±47	19.3±9.1
51	142±19	46±3	126±18	16.6±4.1
82	168±25	54±4	110±42	13.7±4.5
100	130±40	53±4	81±12	9.5±5.4

[a] Measured in compression. [b] Young's modulus. [c] Storage modulus, measured by rotational rheometry. Errors=s.d. with *n*=7–9.

Further examining the viscoelastic properties of the gels by oscillatory rheology, the storage modulus (*G*′) was found to decrease with the % *cis* content (strain ranging from 0.01 % to 10 %, Figure 3 b), in good agreement with the compression data. Specifically, *G*′ increased from 9.5±5.4 kPa to 24.3±0.05 kPa for **4_2A_4_2S_** click‐hydrogels with 100 % and 10 % *cis* content, respectively, which indicates that the click‐hydrogels stiffen as the % *trans* content increases. Moreover, this represents a very large range of stiffness compared to other related systems such as the photoswitchable azobenzene gels[^4^, ^5f^] which are significantly lower (i.e. 10–1000 Pa[^5b^, ^5c^, ^5e^] or between 2 and 10 kPa^[5a]^). The difference in mechanical properties among the stereocontrolled click‐hydrogels is postulated to derive from loop formation within the networks, which results in less perfect networks. With increasing *cis* bond content, the multiarm structures may be more likely to form loops with neighbouring reactive ends that do not participate in the overall network. Hydrogels with higher *cis* content would be envisioned to have more imperfect architectures, thus decreasing the storage modulus of the hydrogel.^[24]^ In contrast, for networks with high *trans* content, the crosslinks allow for greater arm extension and for each multiarm PEG to form an intermolecular crosslink with another PEG unit (Figure S15). This would result in a more perfect network and a more robust, stiffer hydrogel. The GF and EWC values are expected to be unaffected as the loops are still attached to the network.

In good agreement with the mechanical data, the pore size determined from cryo‐SEM images is also evidence of the stiffer nature of the click‐hydrogels with 10 % *cis* content (Figure S16) as the click‐hydrogel network hindered ice growth during the freezing of the sample, which resulted in pores with smaller size (1.9±0.42 μm) in contrast to click‐hydrogels with higher % *cis* content (i.e. 4.4±1.6 μm and 5.0±2.0 μm for 51 % and 100 % *cis* content, respectively). In comparison to mesh size calculations (Table 1), pore size measurements are correlated to ice crystal growth in the click‐hydrogel structures and, therefore, provide information about microscale features. In contrast, mesh size values are derived by taking into account the number of crosslinks in the polymer network at the nanoscale. These pore size measurements suggest that cells exerting forces on the surface of the network would feel a difference in stiffness and hence display a mechanoresponse. Overall, the mechanical performance and stiffness of our **4_2A_4_2S_** click‐hydrogels make them suitable as mimics for several different tissues,^[25]^ such as in the study of fibrosis/fibrotic diseases, cancer, cardiovascular diseases and musculoskeletal diseases (such as osteoarthritis), where interest in elucidating how cells sense the mechanical properties of the matrices they are attached to is involved. Indeed, our approach yielded robust click‐hydrogels with a wide stiffness range (ca. 15 kPa) without varying the crosslinking density or hydrophilicity. Although the resulting click‐hydrogels are still quite rigid (i.e. *G*′ values of 10–25 kPa), click‐hydrogel‐based platforms with overall lower stiffness could be accessible by combining PEG precursors with different molecular weight and architectures to mimic a wider range of biological environments, while retaining the stereochemical tuneability offered by this system.

**Figure 4:**
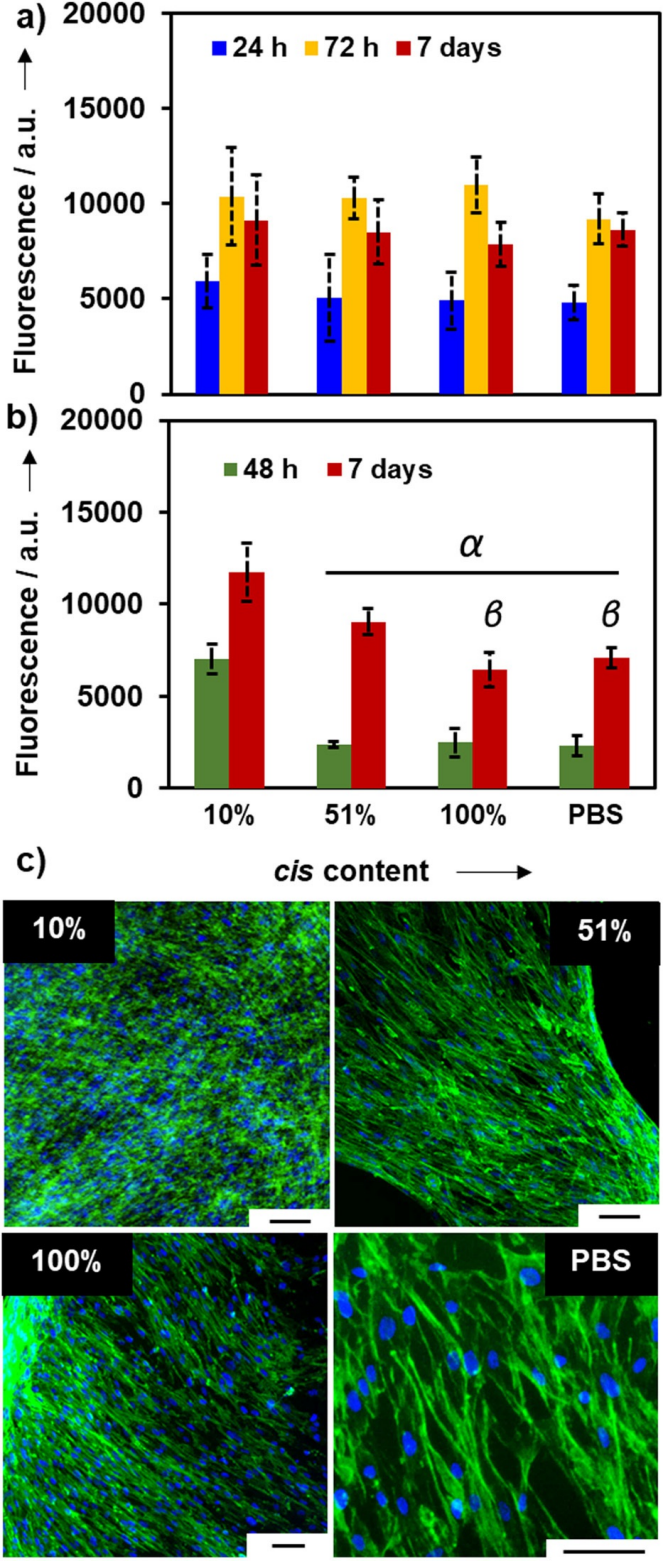
Cytocompatibility of stereocontrolled thiol–yne PEG click‐hydrogels: a) cell viability of MC3T3 cells seeded on top of the click‐hydrogels for 7 days. b) Cell viability of Y201 cells seeded on top of the click‐hydrogels for 7 days. Error bars: SD with *n*=6–12. Greek letters on the bars refer to significant differences within time point groups (*p*‐value<0.05): α vs. 10 %; β vs. 51 %. c) Fluorescence microscopy images of Y201 MSCs on thiol–yne click‐hydrogels at 7‐day time point. Cells in all conditions were fixed at the same time and stained with DAPI (blue) and phalloidin (green). Scale bar=75 μm.

To further investigate the cellular response to the stereochemically controlled stiffness of these **4_2A_4_2S_** click‐hydrogels, we assessed their mechanically induced control over cell responses for potential applications as cell culture platforms. To that end, we selected the click‐hydrogels with a % *cis* content of 10 %, 51 %, and 100 % for further studies, while click‐hydrogels made using PBS as solvent were also included as control. The PBS control was made without the use of TEA and subjected to the same washing and swelling times as the other hydrogels. The PBS gel has also been previously characterised.^[22b]^ All the systems, regardless of the *cis*:*trans* ratio, were determined to be highly cytocompatible. MC3T3 cells (osteoblast precursor cell line derived from mouse calvaria) and human MSCs (Y201 hTERT‐immortalised human clonal MSCs^[26]^) were seeded in a 2D configuration on the top surface of the click‐hydrogels and cultured for 7 d. Cell viability, which was assessed at different time points, was comparable to the control (Figure 4 a,b). After 7 d of incubation, cells formed a dense and compact monolayer on top of the **4_2A_4_2S_** click‐hydrogels, thus resulting in adequate substrates that allowed cell attachment and proliferation (Figure 4 c). Indeed, the stereocontrolled click‐hydrogel‐based platforms exhibited cell viability comparable to that of the control or higher, which was further confirmed by live–dead images (Figure S17). Interestingly, Y201 MSCs tended to grow to a greater extent on stiffer click‐hydrogels after 7 d of incubation (Figure 4 b). We ascribe such response to the fact that a stiffer substrate allows cells to be anchored more strongly than on less stiff substrates, as previously observed.[^5a^, ^27^] Moreover, any leachable products (within 96 h of immersion) from the click‐hydrogels were also determined to be non‐cytotoxic (Figure S17), which confirms the efficacy of the manufacturing process since no remaining solvent or base is left after the washing steps.

**Figure 5:**
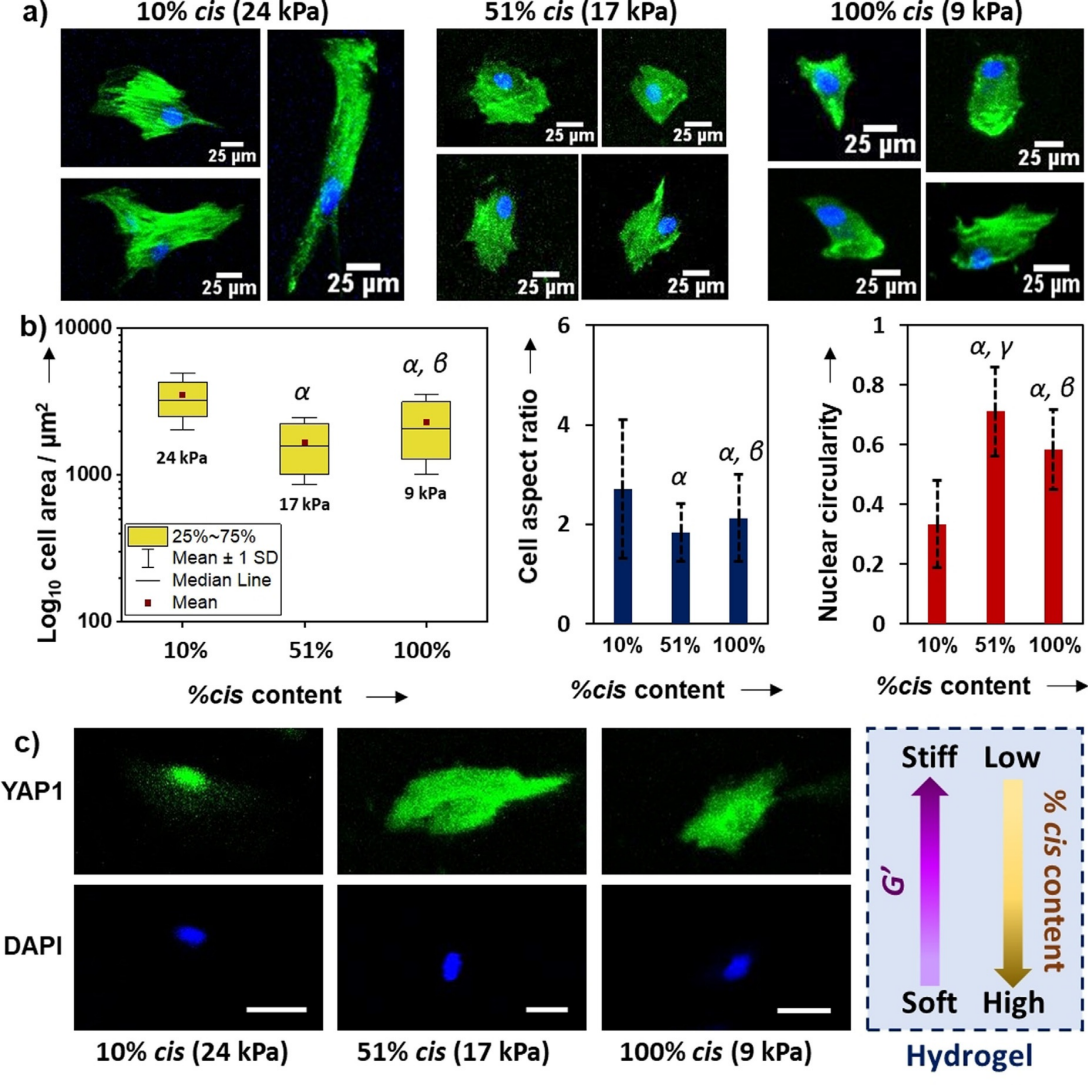
Morphometric data of Y201 MSCs seeded on stereocontrolled thiol–yne PEG click‐hydrogels: a) cell morphology was assessed using phalloidin and DAPI staining following 72 h culture. b) Cell spread area, aspect ratio and circularity data extracted from images. A minimum of 140 immortalised cells were analysed per condition. c) Immunofluorescence images showing transcription factor YAP1 response to stiffness in immortalised MSCs (scale bar 25 μm). Greek letters refer to significant differences (*p*‐value<0.05): α vs. 10 %; β vs. 51 %; and γ vs. 100 % *cis* content.

The immortalised MSC line used (Y201 MSCs), which has been shown to maintain mechanoresponsive behaviour in comparison to primary cells when subjected to modulated substrate mechanics,^[27]^ was selected to be exploited here as a substrate stiffness reporter. Hence, to determine the response of cells to culture on click‐hydrogels with different stiffness, Y201 MSCs were seeded for three days on stiff (10 % *cis* content, *G*′=24.3 kPa) and soft click‐hydrogels (100 % *cis* content, *G*′=9.5 kPa), as well as on substrates with medium stiffness (51 % *cis* content, *G*′=16.6 kPa). After fixing, cell nuclei were stained with DAPI and the actin cytoskeleton with phalloidin (Figure 5 a). As a general trend, cells on stiffer substrates appeared larger and more elongated, whereas cells on softer click‐hydrogels displayed a more circular shape and smaller spread area. Specifically, cell morphology was assessed through quantitative image analysis by measuring cell spread area, cell aspect ratio (defined as the ratio of long to short axis of the smallest rectangle that can enclose the perimeter of a cell), and cell circularity (proportional to the ratio between the area and the square of the perimeter of a cell; ranges from 0 (infinitely elongated polygon) to 1 (perfect circle)). All mean values for cell shape descriptors showed statistically significant differences, which suggests cells responded differently to the stiffness they were exposed to (Figure S18). Specifically, spread cell area decreased from 3523 μm^2^ (stiff click‐hydrogels, 24 kPa, 10 % *cis* content) down to 1669 μm^2^ and 2293 μm^2^ for 51 % (17 kPa) and 100 % *cis* content (9 kPa), which represents a reduction of 53 % and 35 %, respectively (Figure 5 b). Similarly, cell aspect ratio increased with stiffness and varied between 2.7±1.4 (24 kPa, 10 % *cis* content) and 1.8±0.6 (17 kPa, 51 % *cis* content), the value for 100 % *cis* content (9 kPa) being close to the latter (i.e. 2.1±0.8). Therefore, the morphology of Y201 immortalised stem cells was coupled to the substrate stiffness, which was more evident for the stiffest substrate (24 kPa, 10 % *cis* content) where cells spread more. Changes in cellular morphology often propagate to the nucleus because it is physically connected to the cytoskeleton.^[28]^ For instance, nuclear circularity (Figure 5 b) was significantly reduced on stiff substrates (0.33±0.14) in comparison to the two softer substrates (0.71±0.15 and 0.58±0.13, for 51 % (17 kPa) and 100 % *cis* content (9 kPa), respectively). Overall, the morphology of Y201 MSCs, which varied accordingly with the substrate stiffness, was consistent with previous reports.[^5a^, ^27^]

Finally, the subcellular location of Yes‐associated protein 1 (YAP1), which translocates to the nucleus on stiff substrates,^[29]^ was examined with immunofluorescence microscopy in Y201 MSCs cultured on our stereocontrolled thiol–yne click‐hydrogel substrates. After a 7‐day culture period, the distribution of YAP1 appeared to be affected by substrate mechanics (Figure 5 c). Indeed, YAP1 localised to the nucleus of cells seeded on the 10 % *cis* content click‐hydrogels (*G*′=24 kPa), whereas it was observed in the cytoplasm of cells seeded on softer substrates, that is, 51 % (*G*′=17 kPa) and 100 % (*G*′=9 kPa) *cis* content. Therefore, immortalised MSCs exhibited substrate‐directed regulation of YAP1 subcellular location.

Overall, the stereochemical control of the nucleophilic thiol–yne addition reaction afforded click‐hydrogels with adjustable *cis*:*trans* content. The stereochemically controlled hydrogels display analogous physical properties, yet divergent mechanical properties, likely correlated to the amount of loop defects within the networks (Figure S15). As a result of these network imperfections, Y201 MSCs sense varying mechanical features in the network and, consequently, YAP subcellular location is modified.

## Conclusion

We have described how facile modulation of stereochemistry can be achieved without affecting other critical hydrogel characteristics, thus highlighting the unique opportunity that stereochemistry brings to hydrogel synthesis. Most importantly, our approach, which “locked” the *cis*:*trans* ratio during gelation by adjusting solvent polarity and base, resulted in click‐hydrogels with similar crosslinking density, hydrophilicity and swelling/degradation profiles yet, solely as a result of the stereochemistry of the in situ‐formed double bond. These enabled the hydrogels to display large differences in mechanical strength and stiffness. Changes in stiffness were sufficient to be satisfactorily reported by an immortalised human MSC line, which presents the use of stereochemistry to control click‐hydrogel mechanical properties to generate hydrogel substrates that can aid understanding cell responses and other biological processes exclusively as a result of mechanical cues. Overall, we envision our system as a powerful platform to study cell‐microenvironment interactions, mechanotransduction signalling pathways and biological processes. Ultimately, this could advance many technologies related to tissue regeneration, wound healing, embryonic development and also afford additional insight into probing disease progression.^[30]^ Currently, our stereocontrolled thiol–yne click‐hydrogel interfaces are being considered for additional tissue engineering applications. In the future, this chemistry could be used to develop personalised medicine or broaden our understanding into disease progression.

## Conflict of interest

The authors declare no conflict of interest.

## Supporting information

As a service to our authors and readers, this journal provides supporting information supplied by the authors. Such materials are peer reviewed and may be re‐organized for online delivery, but are not copy‐edited or typeset. Technical support issues arising from supporting information (other than missing files) should be addressed to the authors.

Supporting Information
